# Overexpression of Plasma Membrane H^+^-ATPase in Guard Cells Enhances Light-Induced Stomatal Opening, Photosynthesis, and Plant Growth in Hybrid Aspen

**DOI:** 10.3389/fpls.2021.766037

**Published:** 2021-11-26

**Authors:** Shigeo Toh, Naoki Takata, Eigo Ando, Yosuke Toda, Yin Wang, Yuki Hayashi, Nobutaka Mitsuda, Soichiro Nagano, Toru Taniguchi, Toshinori Kinoshita

**Affiliations:** ^1^Department of Environmental Bioscience, Meijo University, Nagoya, Japan; ^2^Division of Biological Science, Graduate School of Science, Nagoya University, Nagoya, Japan; ^3^Forest Bio-Research Center, Forestry and Forest Products Research Institute, Hitachi, Japan; ^4^Department of Biological Sciences, Graduate School of Science, The University of Tokyo, Tokyo, Japan; ^5^Japan Science and Technology Agency, Saitama, Japan; ^6^Institute of Transformative Bio-Molecules (WPI-ITbM), Nagoya University, Nagoya, Japan; ^7^Phytometrics co., ltd., Shizuoka, Japan; ^8^Institute for Advanced Research, Nagoya University, Nagoya, Japan; ^9^Institute of Ecology, College of Urban and Environmental Sciences and Key Laboratory for Earth Surface Processes of Ministry of Education, Peking University, Beijing, China; ^10^Bioproduction Research Institute, National Institute of Advanced Industrial Science and Technology (AIST), Tsukuba, Japan; ^11^Global Zero Emission Research Center, National Institute of Advanced Industrial Science and Technology (AIST), Tsukuba, Japan; ^12^Forest Tree Breeding Center, Forestry and Forest Products Research Institute, Hitachi, Japan; ^13^Tohoku Regional Breeding Office, Forest Tree Breeding Center, Forestry and Forest Products Research Institute, Takizawa, Japan

**Keywords:** PUMP, PM H^+^-ATPase, guard cell, stomatal conductance, hybrid aspen

## Abstract

Stomata in the plant epidermis open in response to light and regulate CO_2_ uptake for photosynthesis and transpiration for uptake of water and nutrients from roots. Light-induced stomatal opening is mediated by activation of the plasma membrane (PM) H^+^-ATPase in guard cells. Overexpression of PM H^+^-ATPase in guard cells promotes light-induced stomatal opening, enhancing photosynthesis and growth in *Arabidopsis thaliana*. In this study, transgenic hybrid aspens overexpressing Arabidopsis PM *H^+^-ATPase* (*AHA2*) in guard cells under the strong guard cell promoter Arabidopsis *GC1* (*AtGC1*) showed enhanced light-induced stomatal opening, photosynthesis, and growth. First, we confirmed that *AtGC1* induces GUS expression specifically in guard cells in hybrid aspens. Thus, we produced *AtGC1::AHA2* transgenic hybrid aspens and confirmed expression of *AHA2* in *AtGC1::AHA2* transgenic plants. In addition, *AtGC1::AHA2* transgenic plants showed a higher PM H^+^-ATPase protein level in guard cells. Analysis using a gas exchange system revealed that transpiration and the photosynthetic rate were significantly increased in *AtGC1::AHA2* transgenic aspen plants. *AtGC1::AHA2* transgenic plants showed a>20% higher stem elongation rate than the wild type (WT). Therefore, overexpression of PM H^+^-ATPase in guard cells promotes the growth of perennial woody plants.

## Introduction

In an era of global climate change and food shortages, finding ways to improve the absorption of CO_2_ by land plants is becoming an increasingly important issue. Stomatal pores in the epidermis are surrounded by two guard cells and are important for capturing CO_2_. Stomata are found mainly on the surface of leaves in land plants. Because the leaf surface is almost impermeable to air and water, the stomatal pores are the primary pathway for diffusion of CO_2_, O_2_, and water vapour between the atmosphere and interior of the leaf (Willmer and Fricker, 1995). Enhancement of gas exchange by stomatal opening is essential for photosynthesis and transpiration (Shimazaki et al., 2007). Stomatal transpiration limits photosynthesis in rice (Kusumi et al., 2012). Therefore, increasing the stomatal opening and transpiration could enhance photosynthesis and thus plant growth. Condon et al. (1987) examined diverse wheat genotypes and found that increasing stomatal conductance, especially abaxial stomatal conduct, enhanced crop biomass. Transgenic *Arabidopsis thaliana* (Arabidopsis) overexpressing plasma membrane (PM) H^+^-ATPase, a key enzyme for stomatal opening, in guard cells promotes light-induced stomatal aperture opening, photosynthetic activity, and plant growth (Wang et al., 2014). Furthermore, the overexpression of PM H^+^-ATPase in rice increases stomatal opening, nutrient uptake, and photosynthesis, thus enhancing grain yield in paddy fields (Zhang et al., 2021). Therefore, we propose designating plants overexpressing PM H^+^-ATPase as Promotion and Upregulation of plasma Membrane Proton-ATPase (PUMP) plants.

Light stimulates the stomatal opening, and there are several mechanisms of stomatal opening in response to light of different wavelengths (Shimazaki et al., 2007; Inoue and Kinoshita, 2017). Blue light is a major stimulator of the stomatal opening. The blue light receptors, phototropins (phot1 and phot2), activate PM H^+^-ATPase in the PM by binding 14-3-3 protein to the phosphorylated penultimate residue, threonine (Thr; Kinoshita and Shimazaki, 1999; Kinoshita et al., 2001). Following activation by blue light, PM H^+^-ATPase induces hyperpolarisation of the PM, allowing K^+^ uptake through inwardly rectifying K^+^ (K^+^_in_) channels (Shimazaki et al., 2007). The accumulation of K^+^ causes guard cells to swell and pores to open. Several signal components – such as blue light signalling 1 (BLUS1), type 1 protein phosphatase, and blue light-dependent H^+^-ATPase phosphorylation (BHP) – mediate blue light-dependent signalling in guard cells (Takemiya et al., 2013; Hayashi et al., 2017). Red light opens stomata by decreasing the intercellular CO_2_ concentration (Ci) and photosynthesis in leaf chloroplasts and stomata (Sharkey and Ogawa, 1987; Roelfsema and Hedrich, 2005; Vavasseur and Raghavendra, 2005). However, the mechanism of the stomatal response to red light is unclear (Baroli et al., 2008; Wang et al., 2011). Red light induces stomatal opening in whole leaves by activating PM H^+^-ATPase *via* photosynthesis-dependent phosphorylation of its penultimate residue, Thr (Ando and Kinoshita, 2018).

Forest trees fix atmospheric CO_2_ mainly into wood biomass. Indeed, forest products, such as timber, contain large amounts of carbon, contributing to mitigation of climate change. *Populus* is one of the fastest growing trees in the Northern Hemisphere and is ideal for furniture, paper pulp, and biofuel production. The genomic sequence of *Populus trichocarpa* was published in 2006 (Tuskan et al., 2006), facilitating transgenic approaches to improving the growth and wood properties of *Populus* species. Enhancement of tree growth and biomass production is typically accomplished by overexpression of endogenous and exogenous *Populus* genes and by RNAi repression of *Populus* endogenous genes (reviewed in Dubouzet et al., 2013). For example, photosynthetic yield and assimilation have been modified to increase plant biomass in *Populus*. In *P. trichocarpa*, the overexpression of *Populus Photoperiod Response 1*, which is associated with starch accumulation, enhances starch accumulation in transgenic plants, thereby increasing biomass production in stem and root (Zawaski et al., 2012). Because the PUMP plant’s strategy is effective in eudicotyledonous and monocotyledonous plants, it may also be useful for enhancing photosynthetic activity and biomass production in perennial woody plants.

In this study, the PUMP plant’s strategy was applied to hybrid aspen (*Populus tremula*×*Populus tremuloides*), a perennial woody plant, to enhance plant growth and biomass production. Overexpression of Arabidopsis PM *H^+^-ATPase* (*AHA2*) under *CaMV35S* promoter could not be achieved in hybrid aspen. Therefore, we used the guard cell-specific promoter Arabidopsis *GC1* (*AtGC1*) to overexpress *AHA2* in hybrid aspen. *AtGC1* was active in guard cells in hybrid aspen as in Arabidopsis. *AtGC1::AHA2* transgenic hybrid aspens showed higher stomatal conductance and photosynthetic rate compared to wild-type (WT) plants. The transgenic plants were taller and had more biomass than WT plants when grown in a greenhouse for 2months. Therefore, the PUMP plant’s strategy can increase growth and biomass production in perennial plants.

## Materials and Methods

### Phylogenetic Tree and Bioinformatics

Plasma membrane *H^+^-ATPase* genes were retrieved from genomic databases for *A. thaliana* (The *Arabidopsis* Information Resource, TAIR) and *P. trichocarpa* (Phytozome v. 12.1). Amino acid sequences were aligned using ClustalW. Evolutionary distances were computed using the Jones-Taylor-Thornton (JTT) matrix-based method with the complete-deletion option (Jones et al., 1992). Phylogenetic trees were constructed by the neighbour-joining (NJ; Saitou and Nei, 1987) and maximum-likelihood (ML) methods. Bootstrap values were calculated with 1,000 replications using the NJ (Felsenstein, 1985) and ML methods in MEGA7 software (Kumar et al., 2016).

Tissue-specific gene expression patterns of 13 *Populus* PM *H^+^-ATPase* genes were examined by re-analysing the RNA sequencing data (Shi et al., 2017). We normalised the raw count data set obtained by RNA sequencing (GSE81077) for xylem, phloem, leaf, shoot, and root with trimmed mean M-values using edgeR v. 3.18.1 (Robinson et al., 2010) in R software v. 3.3.2 (R Core Team, 2018; Hori et al., 2020).

### Plant Materials and Growth

*Populus tremula*×*Populus tremuloides* (WT clone T89) seedlings were cultured in 0.8% (w/v) agar box containing 0.5× Murashige and Skoog medium (pH 5.7) at 25°C under a cycle of 16-h white light (50μmolm^−2^ s^−1^)/8-h dark. Cultured hybrid aspens were transferred to the soil mix (3:1 fertilised peat moss: vermiculite, v/v) and grown in two different conditions. One is a greenhouse, and the other is an indoor plant growth room. Greenhouse temperatures were maintained at 21.5±8°C, and natural light was supplemented with metal halide lamps (KI Holdings, Yokohama, Japan) to achieve an 18-h daylength (PAR≥200μmolm^−2^ s^−1^)/6-h dark. The indoor plant growth was maintained at 20°C, and plants were grown under a cycle of 16-h white light (80μmolm^−2^ s^−1^)/8-h dark. Plants were watered and fertilised once weekly with 2,000-fold diluted Hyponex 6-10-5 solution (HYPONeX Japan, Osaka, Japan) for all conditions.

### Generation of Transgenic Hybrid Aspens

For *AtGC1::GUS* and *CaMV35S::AHA2* constructs, *AtGC1* and *AHA2* were cloned into the pCR8/GW/TOPO vector (Thermo Fisher Scientific, Waltham, MA, United States) and transferred to the pGWB433 and pGWB402 vectors *via* the Gateway LR reaction (Nakagawa et al., 2007). Construction of *AtGC1::AHA2* was described previously (Wang et al., 2014). The binary vectors (pGWB433-*AtGC1::GUS*, pGWB402-*CaMV35S::AHA2*, and pPZP211-*AtGC1::AHA2*) were transformed into *Agrobacterium tumefaciens* strain GV3101 (pMP90). Transgenic hybrid aspens were generated using the vectors, essentially as described by Eriksson et al. (2000).

### GUS Staining of Transgenic Hybrid Aspen

Samples were thoroughly rinsed in distilled water and placed in cold 90% acetone on ice for 5min. Acetone was removed, and GUS staining solution was added for 20min, followed by incubation overnight at 37°C. GUS staining solution consisted of 10mM Na_2_EDTA, 50mM phosphate buffer (pH 7.0), 1mM K_4_Fe(CN)_6_, 1mM K_3_Fe(CN)_6_, 0.5mg/ml X-Gluc (5-bromo-4-chloro-3-indolyl β-D-glucuronide), and 0.1% Triton X-100. Stained samples were soaked in 70% (v/v) ethanol to remove chlorophyll.

### Reverse Transcription PCR for Gene Expression

Total RNA was extracted from epidermal fragments using the NucleoSpin RNA Plant kit (TaKaRa Bio, Shiga, Japan). Epidermal fragments from whole leaves were isolated from 10-week-old plants as described previously (Hayashi et al., 2011). First-strand cDNAs were synthesised from total RNA using the QuantiTect Reverse Transcription Kit (Qiagen, Hilden, Germany). Reverse transcription PCR was performed using 2μl of cDNA template with Ex Taq PCR Mix (TaKaRa Bio) and specific primers. Primers were shown in Supplementary Table 1. Master mix of PCR reaction was prepared for each gene of interest, and 20μl of reaction mix, including the cDNA template, was pipetted into each tube. The conditions were 1cycle at 95°C for 2min; 24 (*UBQ*), 28 (*AHA2* and *Pt×tHA2*) cycles of 95°C for 15s, 57°C for 30s, and 72°C for 30s; and a final incubation at 72°C for 2min. Amplified cDNA was detected by electrophoresis.

### Immunohistochemical Detection of Plasma Membrane H^+^-ATPase in Guard Cells

Immunohistochemical detection was performed as described previously (Hayashi et al., 2011). Polyclonal antibodies against the conserved catalytic domain of the plasma membrane H^+^-ATPase of Arabidopsis (AHA2) were raised in rabbits. The AHA2 DNA fragment was amplified from first-strand Arabidopsis cDNA with PCR using the specific primers 5ʹ-GCCGGATCCATGGATGTCCTGTGCAGTGAC-3ʹ and 5ʹ-GCCGGATCCTCAAGCACCACGAGCAGC-3ʹ. The resulting amplified DNA fragment of 967–1,845bp of AHA2, which contains BamHI sites at both ends, was cloned into the BamHI site of the pET30a vector (Merck, Darmstadt, Germany). The purified proteins from *E. coli* (BL21) were used as an antigen. The antiserum was used for immunoblots in Arabidopsis (1:1,000 dilution; Hayashi et al., 2010). PM H^+^-ATPase was detected in guard cells using epidermis isolated from hybrid aspen leaf. The amount of PM H^+^-ATPase was estimated using antiserum against the catalytic domain of AHA2. Fluorescence intensity was quantified according to Ando and Kinoshita (2018).

### Gas Exchange Measurements

Gas exchange measurements were performed as described previously (Wang et al., 2014) using the LI-6400 system (Li Cor Biosciences, Lincoln, NE, United States), and parameters were calculated with the software supplied by the manufacturer. White light (1,000μmol·m^−2^·s^−1^) was provided by a fibre optic illuminator with a halogen projector lamp (15V/150W; Moritex, Saitama, Japan) as a light source and a MHAB-150W; power supply (Moritex). Light was attenuated by a series of optical crown glass metallic neutral density filters (Newport Japan, Hakuto, Japan). The molar flow rate of air entering the leaf chamber, leaf temperature, and relative humidity was maintained at 500μmol·s^−1^, 24°C, and 40–50% (Pa/Pa), respectively. After the initial 10min of dark adaptation, the plants were exposed to white light (1,000μmol·m^−2^·s^−1^) for 30min.

### Growth Analyses and Biomass Assays

Plant height was measured weekly from 21days after potting in a greenhouse. Once trees had reached 20cm in height, the stem diameters were measured weekly at 10cm above the soil. The elongation growth rate of plants was evaluated by a curve-fitting procedure (Buchwald, 2007; Edwards et al., 2018). The radial growth rate was calculated by fitting to a linear function. These procedures were conducted in KaleidaGraph v. 4.1 (Synergy Software, Reading, PA, United States). Leaf number and size (leaves 16–25) were measured when sampling leaves. Leaves were imaged using a scanner (Perfection V700 Photo; Epson, Nagano, Japan) at 600dpi, and leaf size was evaluated by ImageJ 1.51 software.^1^ Leaves, stems, and roots were collected from each plant and weighed to calculate the fresh weight. Following 3days of drying at 60°C, the leaves, stems, and roots were weighed again to determine the dry weight (DW). The index of stem volume (volumetric index) was calculated as (diameter ÷ 2)^2^×height×π, from the final diameter (cm) and height (cm) of an individual tree. A 1cm length of stem segment was sampled from 2cm above the soil to determine wood density. Xylem tissues were obtained by peeling off the bark and were then filled with ultrapure water. The weight increase by increased water volume (V) was measured by a balance at 20°C. The xylem samples were dried in an oven at 105°C for 72h, and DW was measured using a balance. The wood density was calculated by the formula: Wood density=DW ÷ V.

### Statistical Analysis

Statistical significance was evaluated by Student’s *t* test followed by the multiple testing correction procedure of Benjamini and Hochberg (1995), performed using Excel (Microsoft Corp., Redmond, WA, United States).

## Results

### Phylogeny and Protein Structure of PM H^+^-ATPase Homologs in *Populus*

**Figure 1:**
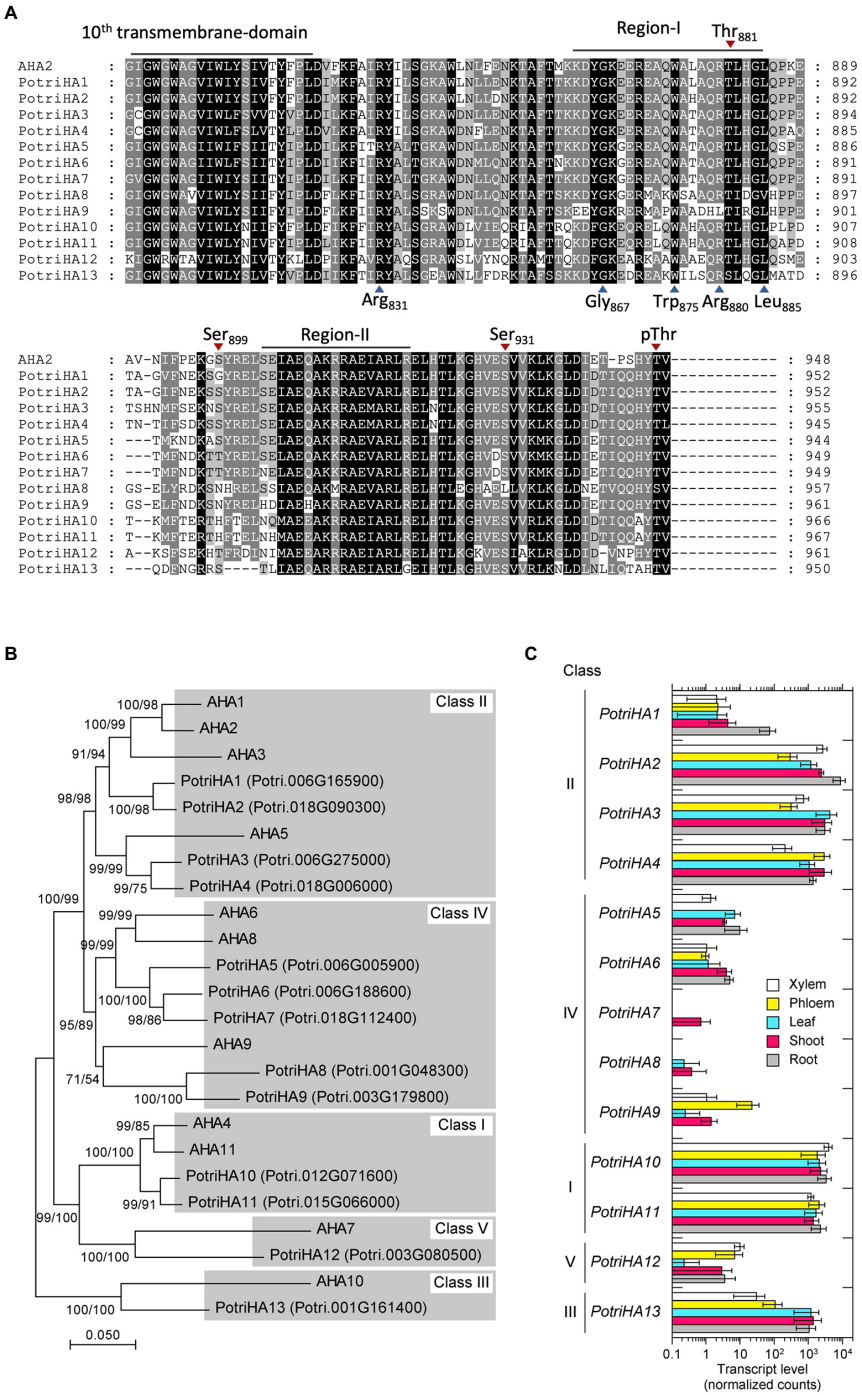
Amino acid sequence similarity and gene expression of *Populus PM H^+^-ATPases*. **(A)** Amino acid sequence alignment of *P. trichocarpa* H^+^-ATPases with the C-terminal inhibition domain of Arabidopsis PM *H^+^-ATPase* (AHA2). The 10th transmembrane domain and the inhibitory motifs (regions I and II) in the C-terminal inhibitory domain are shown. Identical and similar amino acid residues are highlighted by black and grey backgrounds, respectively. Blue arrowheads below the sequence alignment indicate amino acids important for the function of the inhibitory domain of AHA2 (Axelsen et al., 1999) Red arrowheads above the sequence alignment indicate phosphorylation target sites of AHA2 (Fuglsang et al., 2007; Niittylä et al., 2007; Haruta et al., 2014). **(B)** Phylogenetic tree of PM H^+^-ATPases in *A. thaliana* and *P. trichocarpa*. Phylogenetic trees were reconstructed by the neighbour-joining (NJ) and maximum-likelihood (ML) methods based on the alignment of full-length amino acid sequences. The phylogenetic topology was the same in trees reconstructed by the NJ and ML methods. Bootstrap values were calculated by the NJ method with 1,000 replications (left) and by the ML method with 1,000 replications (right). Roman numerals indicate classes, as defined by Arango et al. (2003). **(C)** The expression pattern of *P. trichocarpa H^+^-ATPase*s in xylem, phloem, leaf, shoot, and root tissues. The raw count data set obtained by tissue-specific RNA sequencing (GSE81077, Shi et al., 2017) was reanalysed to calculate normalised read counts as gene expression level of each gene. Error bars represent the SD with three sample replicates.

The *P. trichocarpa* genome has 13 PM H^+^-ATPase (HA) homologs with high amino acid similarity to *A. thaliana* HA2 (AHA2; Figure 1; Supplementary Table 2). We designated the *P. trichocarpa* isoforms PotriHA1–PotriHA13. The *Populus* isoforms have a highly conserved characteristic sequence, GDGVNDAPALKKA, in the catalytic domain of the P-type ATPase (Axelsen and Palmgren, 1998), supporting our proposal that these isoforms are functional homologs in *Populus*. The C-terminal region of PM H^+^-ATPases is important for catalytic regulation (Palmgren, 2001; Haruta et al., 2015; Falhof et al., 2016; Inoue and Kinoshita, 2017). All *Populus* isoforms conserve regions I and II, which are important for autoinhibition, in the C-terminal region (Axelsen et al., 1999), and Thr as a penultimate residue, which is important for its activation *via* phosphorylation (Figure 1A). Several phosphorylation sites in the C-terminal domain (Thr-881, Ser-899, and Ser-931), in addition to Thr as a penultimate residue, are also highly conserved in *Populus* PM H^+^-ATPases.

Phylogenetic analysis using full-length amino acid sequences showed that PotriHAs were classified into classes I–V (Figure 1B), as defined in Arango et al. (2003). PotriHA10 and PotriHA11 formed a clade with AHA4 and AHA11 in class I. PotriHA1, PotriHA2, PotriHA3, and PotriHA4 formed a clade with AHA1, AHA2, AHA3, and AHA5 in class II. PotriHA13 formed a clade with AHA10 in class III. PotriHA5, PotriHA6, PotriHA7, PotriHA8, and PotriHA9 formed a clade with AHA6, AHA8, and AHA9 in class IV. PotriHA12 formed a clade with AHA7 in class V. PM H^+^-ATPases in class II, including *A. thaliana* AHA1 and AHA2 and rice OSA7, have a major role in plants (Haruta et al., 2010; Toda et al., 2016). In *P. trhichocarpa*, *PotriHA2*, *PotriHA3*, and *PotriHA4* (class II) showed higher expression than the other class genes in xylem, phloem, leaf, shoot, and root (Figure 1C), suggesting that those isoforms are major PM H^+^-ATPases in *Populus* species.

### Overexpression of *AHA2* Under Arabidopsis *GC1* Promoter in Hybrid Aspen

**Figure 2:**
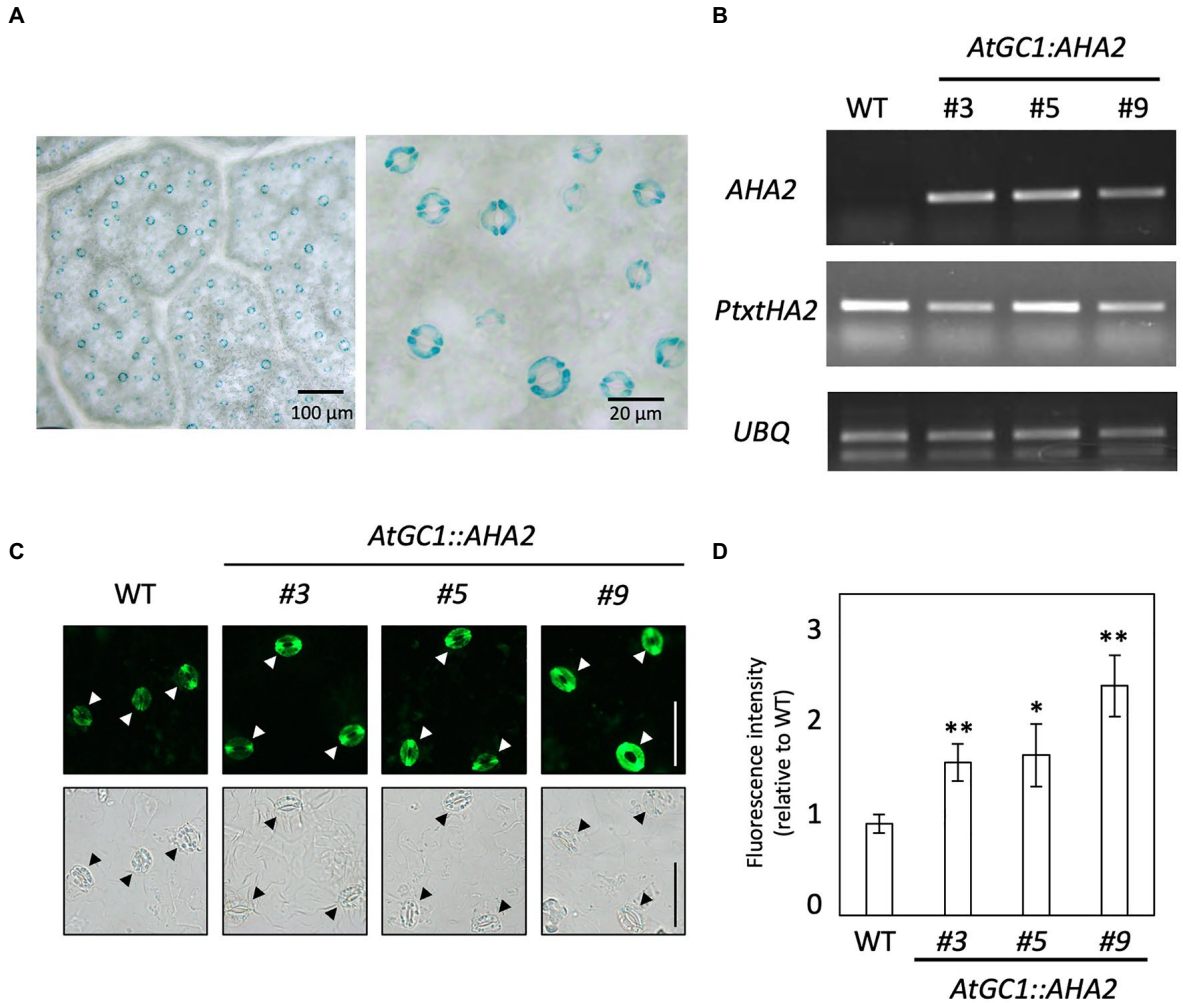
Promoter activity of *AtGC1* in hybrid aspen and generation of *AtGC1::AHA2* transgenic hybrid aspens. **(A)** Histochemical GUS analysis of *AtGC1::GUS* transgenic hybrid aspens. Images are of the abaxial side of the leaf. A high-magnification image is shown in the right panel. Scale bar=100μm (left panel) and 20μm (right panel). **(B)** Expression level of *AHA2* and *P. tremula*×*P. tremuloides* (*Pt×t*) *H^+^-ATPase* in transgenic hybrid aspens and wild type (WT). The expression of *AHA2* and *Pt × tHA2* was analysed by reverse transcription PCR. *Ubiquitin 11* (*UBQ*, Takata et al., 2009) was used as the internal control. **(C)** Immunohistochemical analysis of PM H^+^-ATPase in guard cells of transgenic hybrid aspens and WT. Isolated abaxial leaf epidermis was immunolabeled with antiserum raised against the catalytic domain of AHA2. Fluorescence (upper panel) and bright-field images (lower panel) were captured by a fluorescence microscope. Arrowheads indicate guard cells. Scale bar=50μm. **(D)** Immunofluorescence intensity in guard cells of transgenic hybrid aspens and WT. Fluorescence intensities in transgenic plants were normalised to those in WT plants. Data are means±SD of three independent measurements. Asterisks denote a mean significantly higher than the WT (set to 1.0; Student’s *t* test followed by the Benjamini and Hochberg multiple test correction; ^**^p<0.01 and ^*^p<0.05).

We first attempted to introduce *CaMV35S::AHA2* into hybrid aspen to ectopically overexpress *AHA2*. However, no transgenic hybrid aspens were generated, even using 109 stem segments for *Agrobacterium*-mediated transformation. Therefore, we used the guard cell-specific promoter in *A. thaliana*, *AtGC1*, to express *AHA2* in hybrid aspen. To investigate *AtGC1* activity in hybrid aspen, we generated *AtGC1::GUS* transgenic plants and examined their GUS activity. As shown in Figure 2A, GUS staining was observed in guard cells of the leaf epidermis of *AtGC1::GUS* transgenic plants, similar to *AtGC1::GUS*-expressing *A. thaliana* (Yang et al., 2008). We next transformed the *AtGC1::AHA2* construct into hybrid aspen to overexpress *AHA2* in guard cells and generated at least three independent transgenic events (#3, #5, and #9; Figure 2B). In the transgenic plants, *AHA2* was expressed in leaf epidermis, as was *Pt×tHA2*, a major PM H^+^-ATPase in *Populus*. To examine whether the introduction of *AtGC1::AHA2* elevated the PM H^+^-ATPase protein level in guard cells, immunohistochemical analysis using an anti-AHA2 antibody was conducted in the leaf epidermis of transgenic and WT plants. Immunofluorescence was brighter in guard cells of *AtGC1::AHA2* transgenic plants than WT plants (Figures 2C,D). Fluorescence intensity relative to the WT showed that the protein level of PM H^+^-ATPase was enhanced in guard cells of transgenic plants (70% for #3, 75% for #5, and 150% for #9), indicating that *AtGC1::AHA2* transgenic plants over-accumulated PM H^+^-ATPase in guard cells. The density of stomata in *AtGC1::AHA2* transgenic plants was comparable to that in WT plants (196 stomata mm^−2^ for WT, 203 for #3, 217 for #5, and 203 for #9; Supplementary Table 3). Therefore, the introduction of *AtGC1::AHA2* increased its protein levels in guard cells without affecting stomatal development in hybrid aspen, similar to *A. thaliana* expressing *AtGC1::AHA2* (Wang et al., 2014).

### Stomatal Conductance and Photosynthetic Rate in *AtGC1::AHA2* Transgenic Hybrid Aspens

**Figure 3:**
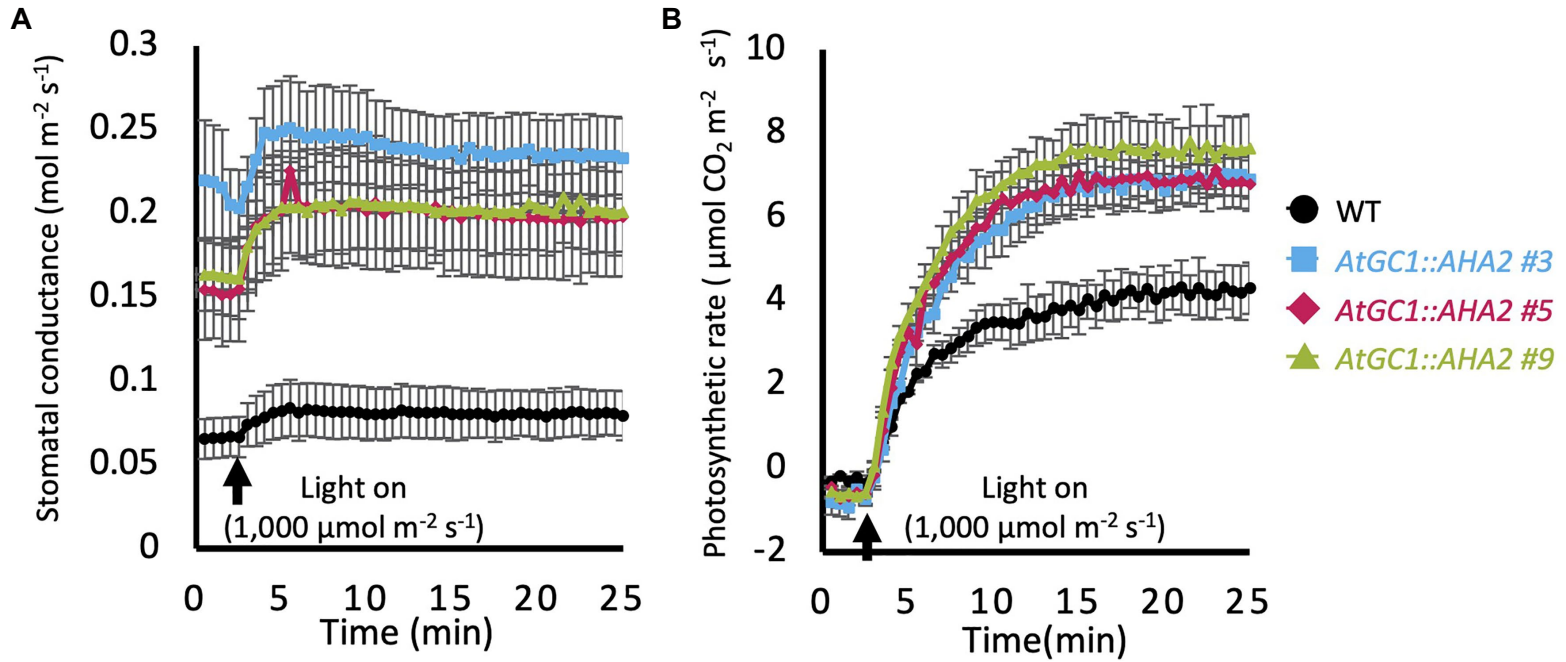
Gas exchange properties of *AtGC1::AHA2* transgenic and wild-type (WT) plants. **(A)** Light responses of stomatal conductance and **(B)** the photosynthetic rate in transgenic and WT plants. Measurements were conducted under dark conditions followed by 1,000μmol.m^−2^.s^−1^ light. Black arrows indicate the time of light-on. Data were plotted every 30s. Measurements were conducted on three different plants for each transgenic event. Error bars represent SE and are not shown if smaller than the symbols.

To investigate photosynthetic activity in *AtGC1::AHA2* transgenic plants, stomatal conductance and the photosynthetic rate (CO_2_ assimilation rate) were measured in intact leaves of transgenic and WT plants grown in an indoor-growth room for 82–94days. The *AtGC1::AHA2* transgenic plants showed higher stomatal conductance in the dark compared to the WT (0.07 for WT, 0.22 for #3, 0.15 for #5, and 0.16 for #9mol·m^−2^·s^−1^. In the WT, white light at 1,000μmol·m^−2^·s^−1^ increased stomatal conductance. Similarly, light illumination increased stomatal conductance in *AtGC1::AHA2* transgenic plants. Stomatal conductance was saturated within 10min of the start of light illumination in the transgenic and WT plants. The average stomatal conductance in the transgenic plants was approximately 3-fold higher than in the WT (Figure 3A). Under identical conditions, photosynthetic rates were saturated 20min after the start of light illumination in WT and *AtGC1::AHA2* transgenic plants. The photosynthetic rate was 45% higher in the transgenic compared to the WT plants (Figure 3B). Although stomatal aperture is used to estimate stomatal conductance and photosynthetic activity, determining the average stomatal aperture is more problematic in hybrid aspen compared to *A. thaliana*, because stomatal size varies in the abaxial epidermis of the former (Supplementary Figure 1; Figure 2A). Taken together, our results indicate that the introduction of AHA2 protein to guard cells increased stomatal conductance and the photosynthetic rate in hybrid aspen.

### Growth Phenology and Biomass Production of *AtGC1::AHA2* Transgenic Hybrid Aspens

**Figure 4:**
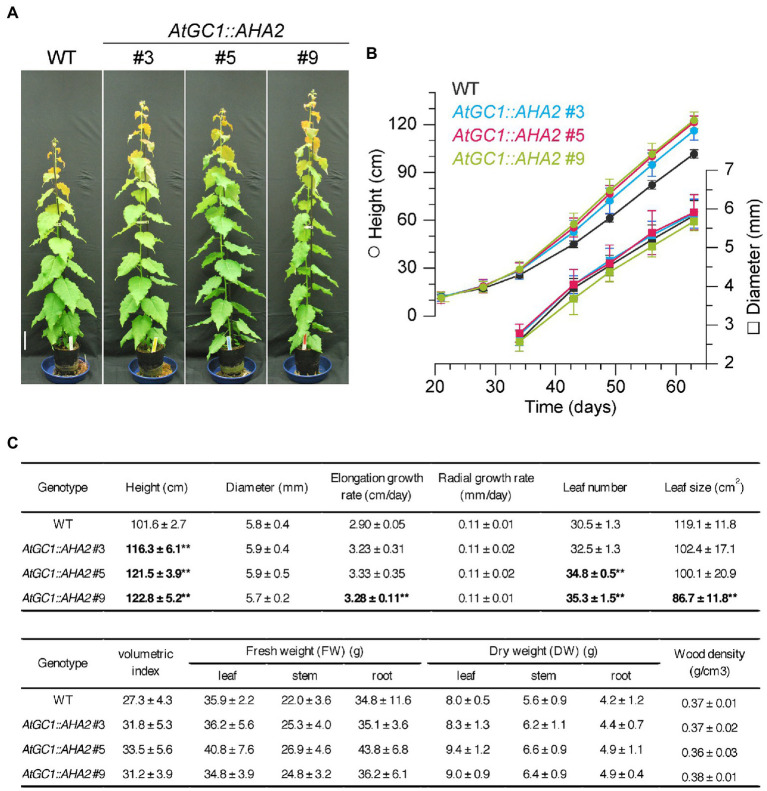
Growth and biomass production of *AtGC1::AHA2* transgenic and wild-type (WT) plants. **(A)** Growth phenotypes of 63-day-old transgenic and WT plants. Scale bar=10cm. **(B)** Height (circles) and diameter (squares) of the transgenic and WT plants against time over 63days of growth. **(C)** Growth rates and biomass production of transgenic and WT plants. Values are means±SD (*n*=4). Double asterisk indicates *p*<0.01 by Student’s *t* test followed by the Benjamini and Hochberg multiple test correction.

Enhancement of photosynthetic activity in *AtGC1::AHA2* transgenic plants was expected to promote growth and biomass production. When *AtGC1::AHA2* transgenic plants and WT plants were grown in a greenhouse for 63days, the transgenic plants showed 14–21% greater height compared to the WT (Figure 4). The elongation rates were 11–15% higher in transgenic than WT plants. The stem diameter and radial growth rates were similar between the transgenic and WT plants. The leaf number per plant was increased 7–16% in the transgenic compared to WT plants, although the area of mature leaves was decreased 14–28%. For biomass production, the volumetric index of stem-trunk biomass was enhanced 14–23% in the transgenic compared to WT plants. Furthermore, the DW of leaves, stems, and roots was non-significantly increased in the transgenic compared to WT plants. However, the stem wood density of the transgenic plants (0.37±0.02g·cm^−3^ for #3, 0.36±0.03g·cm^−3^ for #5, and 0.38±0.01g·cm^−3^ for #9) was similar to that of WT plants (0.37±0.01g·cm^−3^). The increment of tree height in the transgenic plants was observed in the indoor-growth room (Supplementary Figure 2), indicating that the enhancement of growth phenology was stable under different light intensities. Together, our results indicate that *AtGC1::AHA2* transgenic hybrid aspens had a higher stem elongation rate and greater biomass production than the WT, likely due to the enhanced stomatal opening and photosynthetic rate.

## Discussion

In this study, the PUMP plant’s strategy was used to enhance the photosynthetic rate and growth of *Populus* species. PM H^+^-ATPases are highly conserved among plant species, and their gene numbers vary among plant species (e.g., 11 isoforms in *A. thaliana*, nine in *Nicotiana plumbaginifolia*, and 10 in *O. sativa*; Arango et al., 2003). In the genome of *P. trichocarpa*, there were 13 PM H^+^-ATPases, PotriHA1–PotriHA13, with high similarity to *A. thaliana* PM H^+^-ATPase (Figure 1). All isoforms in *P. trichocarpa* had domains typical of plant PM H^+^-ATPases. We overexpressed Arabidopsis *AHA2* under the control of *CaMV35S* or *AtGC1* promoter in hybrid aspens. However, we could not generate *CaMV35S::AHA2* plants. *AtGC1* was specifically expressed in guard cells of hybrid aspens (Figure 2A), indicating that we developed a stomatal-specific promoter in *Populus* species. The *AtGC1::AHA2* transgenic hybrid aspens showed enhanced light-induced stomatal opening (Figure 3). This suggests that PM H^+^-ATPase is the limiting factor in stomatal opening in *Populus* species, as in *A. thaliana* (Wang et al., 2014). Furthermore, the *AtGC1::AHA2* transgenic hybrid aspens had an enhanced photosynthetic rate and growth (Figures 3, 4), indicating that the PUMP plant’s strategy is applicable for perennial trees using a guard cell-specific promoter and PM H^+^-ATPase.

The *AtGC1::AHA2* transgenic hybrid aspens had enhanced stomatal conductance and photosynthetic rate compared to WT plants, increasing plant height, volumetric index, and stem biomass production (Figure 4). The number of leaves and leaf biomass also increased in the transgenic hybrid aspens as elongation growth accelerated, whereas the size of leaves decreased. However, the stem diameter and wood density of the transgenic plants were comparable to those of WT plants. These phenotypic changes imply that improvement of the photosynthetic rate (Figure 3) increased assimilation products, resulting in morphological changes in different tissues of *AtGC1::AHA2* transgenic plants. The allocation of assimilation products varies depending on, for instance, the plant species, plant size, environment, and season. In young cottonwood trees (*Populus deltoides*), younger middle leaves transport assimilation products acropetally and basipetally, while older bottom leaves transport them primarily to lower stem and roots in the growing season (Dickson, 1989). In the present study, hybrid aspens were grown for 2months in a greenhouse and maintained rapid elongation growth. This implies that the increased assimilation products in *AtGC1::AHA2* transgenic hybrid aspens may have been used more for elongation than radial growth in young trees. Because trees grow for many years, develop many branches, and form a large trunk, further study is needed to examine whether carbon allocation changes seasonally and with age in *AtGC1::AHA2* transgenic plants and whether enhancement of the photosynthetic rate by the PUMP plant’s strategy improves biomass production over several years.

The *AtGC1::AHA2* transgenic aspen plants showed basically higher stomatal conductance under both the dark and light than those in WT plants (Figure 3A). It should be noted that enhancement of stomatal opening basically decreases water-use efficiency (WUE). Then, we calculated intrinsic WUE (iWUE; the ratio of photosynthetic rate to stomatal conductance; μmol CO_2_/μmol H_2_O) based on the data from Figure 4 (Leakey et al., 2019). The results showed that iWUE values of WT, *AtGC1::AHA2* transgenic plants #3, #5, and #9 were 52.6, 28.4, 34.4, and 38.4, indicating that the iWUE in *AtGC1::AHA2* transgenic plants was 27–46% lower than that in WT. These results suggest that the *AtGC1::AHA2* transgenic plants enhanced water consumption and required much more water than WT for normal condition. In the case of *A. thaliana*, the *AtGC1::AHA2* transgenic plants showed normal sensitivity to plant hormone abscisic acid (ABA), an inducer of stomatal closing (Wang et al., 2014). Taken together, these results suggest that stomata in the *AtGC1::AHA2* transgenic aspen plants may also show normal sensitivity to ABA and drought responses.

We introduced *Arabidopsis AHA2*, as a typical plant PM H^+^-ATPase, to hybrid aspen. *AtGC1::AHA2* transgenic hybrid aspens showed higher stomatal conductance compared to WT plants in the dark (Figure 3). Enhancement of stomatal opening decreases WUE, indicating that the *AtGC1::AHA2* transgenic hybrid aspens require more water than WT plants for normal growth. Arabidopsis and rice overexpressing endogenous PM *H^+^-ATPase* do not show higher stomatal opening under dark conditions (Wang et al., 2014; Zhang et al., 2021). These results suggest that post-translational modification of Arabidopsis AHA2 in response to light is not fine-tuned in the *AtGC1::AHA2* transgenic hybrid aspens. Because *Populus* HA1, HA2, HA3, and HA4 have high similarities to Arabidopsis AHA2 (Figure 1), overexpressing *Populus* endogenous PM *H^+^-ATPase* in class II may overcome this problem. Further study is needed to generate transgenic hybrid aspen expressing *Populus HA*s under *AtGC1* and to characterise the stomatal properties, light requirements, drought tolerance, and mechanical resilience of the transgenic plants.

Improving the efficiency of photosynthesis can enhance biomass yield. In addition to regulation of stomatal opening, other factors determine the photosynthetic uptake of CO_2_ by plants. Examples include the photosynthetic machinery, carbon flux, photorespiration, photoinhibition, assimilation partitioning, and assimilation utilisation (Dubouzet et al., 2013). Rubisco evolved under conditions characterised by much higher CO_2_ levels than the current ones (Whitney et al., 2011). Therefore, many plants thrive at higher CO_2_ concentrations (Smith et al., 2013). In a study of a deciduous forest, carbon enrichment increased photosynthesis by >40% (Bader et al., 2010). Also, free-air CO_2_ enrichment (FACE) in field plots increased biomass yield by 15–27% in three *Populus* species (Calfapietra et al., 2003). In this study, we promoted light-responsive stomatal opening in hybrid aspens. Thus, our results are consistent with the growth-promoting effect of CO_2_ concentration. Synergistic effects may be obtained by combining these growth conditions with PUMP plants.

We used *AtGC1* for expression of PM H^+^-ATPase in guard cells. The *AtGC1::AHA2* transgenic hybrid aspens showed superior growth for ≥2months after potting. However, we did not investigate plant phenotypes over the long-term. Furthermore, we grew plants in a greenhouse or indoor plant growth room, so plant growth in the field is unknown. We are planning long-term field experiments to verify the usefulness of the PUMP plant’s strategy in perennial woody plants. In addition, we produced *AtGC1::AHA2* transgenic plants; in future, when PM H^+^-ATPase overexpression or activation can be achieved by non-transgenic techniques – for example, genome editing and chemical treatments – such plants could enhance tree biomass.

## Data Availability Statement

The original contributions presented in the study are included in the article/Supplementary Materials, further inquiries can be directed to the corresponding authors.

## Author Contributions

ST, NT, YT, YW, NM, TT, and TK designed the experiments. ST, NT, EA, YT, YW, YH, and SN performed the experiments. ST, NT, and TK wrote the manuscript. NM and TK contributed to the original idea of the project and supervised the study and prepared the manuscript. All authors contributed to the article and approved the submitted version.

## Funding

This work was supported in part by the Advanced Low Carbon Technology Research and Development Program of the Japan Science and Technology Agency (JPMJAL1011 to TK; JPMJAL1107 to NM and TT) and Grants-in-Aid for Scientific Research on Innovative Areas (20H05687 and 20H05910 to TK).

## Conflict of Interest

YT was employed by Phytometrics, co., ltd.

The remaining authors declare that the research was conducted in the absence of any commercial or financial relationships that could be construed as a potential conflict of interest.

## Publisher’s Note

All claims expressed in this article are solely those of the authors and do not necessarily represent those of their affiliated organizations, or those of the publisher, the editors and the reviewers. Any product that may be evaluated in this article, or claim that may be made by its manufacturer, is not guaranteed or endorsed by the publisher.

## Footnotes

^1^ https://imagej.net/
