# Supplementary material for: Overexpression of Plasma Membrane H+-ATPase in Guard Cells Enhances Light-Induced Stomatal Opening, Photosynthesis, and Plant Growth in Hybrid Aspen
Source: Front Plant Sci. 2021 Nov 26;12:766037. doi: 10.3389/fpls.2021.766037 (PMC8663642; doi:10.3389/fpls.2021.766037)
Supplement: Supplementary file 1 [file Data_Sheet_1.pdf]

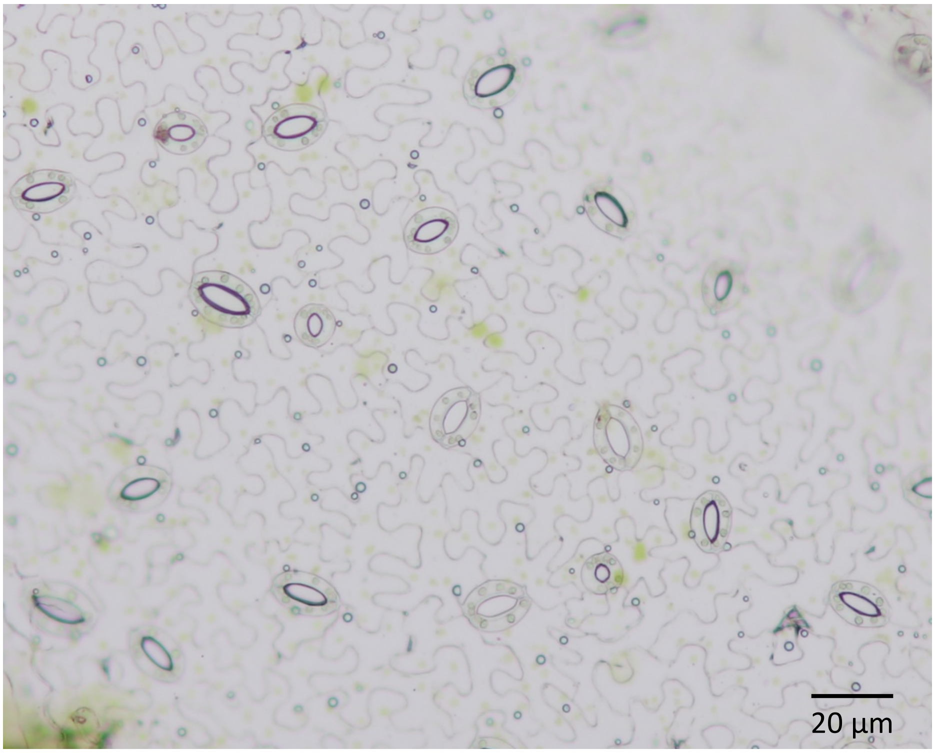

Supplementary Figure 1. Abaxial leaf epidermis of hybrid aspens. The variation in size of epidermal pores prevented quantification of the stomatal aperture.

Supplementary Figure 2

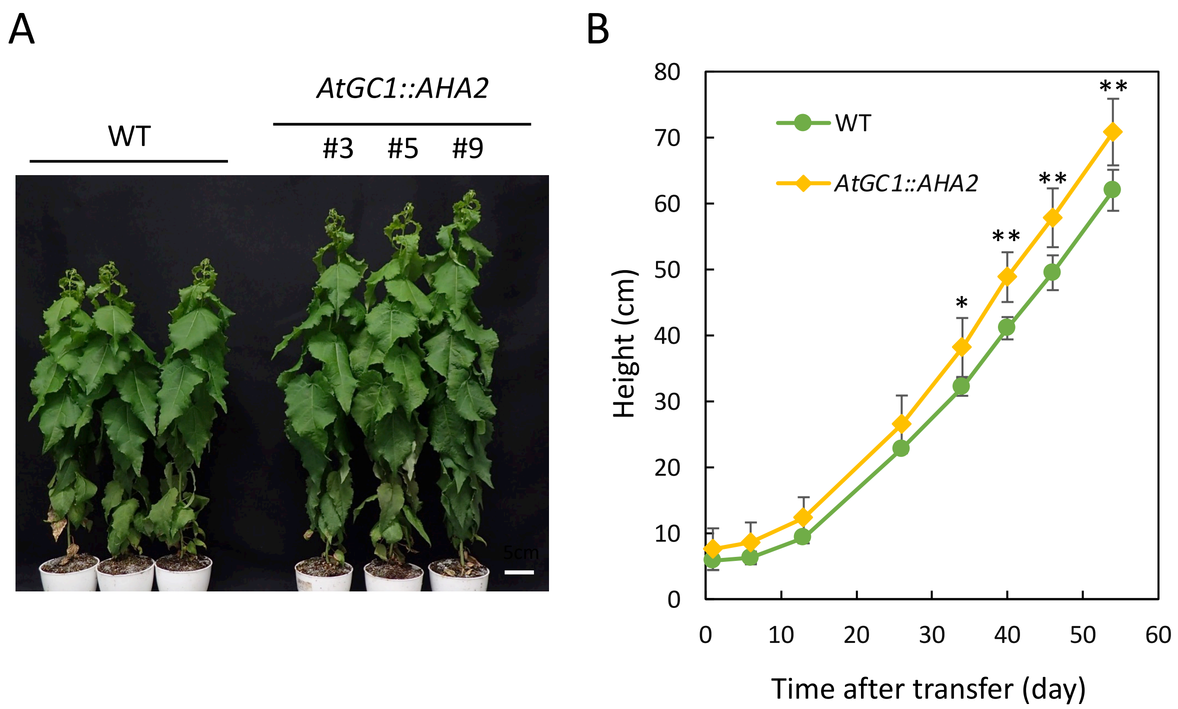

Supplemental Figure 2. Growth of *AtGC1::AHA2* transgenic and wild-type (WT) plants in the indoor plant growth room. (A) Phenotypes of 46-day-old *AtGC1::AHA2* transgenic and WT plants. (B) Height of transgenic and WT plants against time over 54 days of growth. Data are means of three *AtGC1::AHA2* transgenic plants. Each transgenic line has two individual plants. Error bars represent SD and are not shown if smaller than the symbols. Asterisks denote a mean significantly higher than the WT (two-tailed *t*-test; $^{*}P < 0.05$, $^{**}P < 0.01$).

Supplementary Table 1. Sequences of primers for reverse transcription PCR.

Gene		Sequence
AHA2	Fw	TTGTTGAACGTCCTGGAGCA
	Rv	CTACACAGTGTAGTGACTGGGAG
UBQ	Fw	GGTTGATTTTTGCTGGGAAGC
	Rv	TTAAGGCTTGCAATGGAATGG
PtxtHA2	Fw	TAATGTCTGCTTTCATGATTGC
	Rv	GGGTTTGTCATTGAACTTTAGG

Supplementary Table 2. Comparison of *Populus trichocarpa* PM H^+-ATPase isoforms with AHA2.

	Identity (%)
PotriHA1	85.73%
PotriHA2	82.38%
PotriHA3	83.10%
PotriHA4	82.38%
PotriHA5	80.71%
PotriHA6	80.33%
PotriHA7	79.92%
PotriHA8	76.82%
PotriHA9	78.49%
PotriHA10	78.15%
PotriHA11	77.79%
PotriHA12	73.64%
PotriHA13	75.34%

Supplementary Table 3. Stomatal density in WT and *AtGC1::AHA2* transgenic hybrid aspens.

Genotype	Density (number of stomata per mm^2)	SD	*p* value
WT	196.3	± 18.1	
AtGC1:AHA2 #3	203.5	± 33.9	0.763
AtGC1:AHA2 #5	217.8	± 18.1	0.218
AtGC1:AHA2 #9	203.5	± 33.2	0.758

Stomatal density was counted in abaxial epidermis from mature leaves.
Date represent means of densities in 3 different leaves. *p* value were calculated using Student's *t* test (two-sided) compared with WT and each transgenic hybrid aspens.
